# Reducing the rate of guideline-discordant therapy for inpatients with community-acquired pneumonia

**DOI:** 10.1017/ash.2023.244

**Published:** 2023-09-29

**Authors:** Kellie Arensman Hannan, Paul Frykman, Eric Mathiowetz, Jill Sathre, Nou Cheng Yang, Kelsey Jensen

## Abstract

**Background:** Despite guidelines recommending shorter durations of therapy and empiric coverage of *Pseudomonas aeruginosa* and methicillin-resistant *Staphylococcus aureus* (MRSA) only for patients with certain risk factors, optimizing therapy for community-acquired pneumonia (CAP) remains a challenge for antimicrobial stewardship (AMS) teams. We investigated the impact of a multimodal AMS initiative on the rate of guideline-discordant empiric antibiotic selection and total duration of therapy for CAP. **Methods:** A quality improvement initiative was implemented at 9 community hospitals in 2022 to optimize CAP therapy. Education was provided to pharmacists and providers. Alerts were implemented within the electronic medical record to prompt the AMS team to review fluoroquinolones, antipseudomonal β-lactams, and anti-MRSA agents ordered for CAP. Clinical pharmacists reviewed antibiotic orders for CAP at hospital discharge and encouraged providers to prescribe a total antibiotic duration of 5–7 days. For the preintervention period (July– September 2021) and the postintervention period (July to September 2022), a random sample of 320 patients with an antibiotic order for CAP were evaluated retrospectively via chart review. Patients treated for an indication other than CAP were excluded. The primary outcome was the proportion of patients with a total duration of therapy >7 days. Secondary outcomes included average duration of therapy, rate of guideline-discordant empiric therapy, and type of guideline discordance. **Results:** In total, 317 patients were included. The proportion of patients with a total duration of therapy >7 days decreased from 29% to 14% (*P* < .01). Average duration of therapy and guideline-discordant empiric therapy also decreased significantly (Table 1). **Conclusions:** This multifaceted AMS initiative was associated with decreased guideline-discordant empiric therapy and decreased total duration of therapy for CAP.

**Disclosures:** None

